# Supplementary figures and images for: High frequency of transition to transversion ratio in the stem region of RNA secondary structure of untranslated region of SARS-CoV-2
Source: PeerJ. 2024 Apr 22;12:e16962. doi: 10.7717/peerj.16962 (PMC11044879; doi:10.7717/peerj.16962)

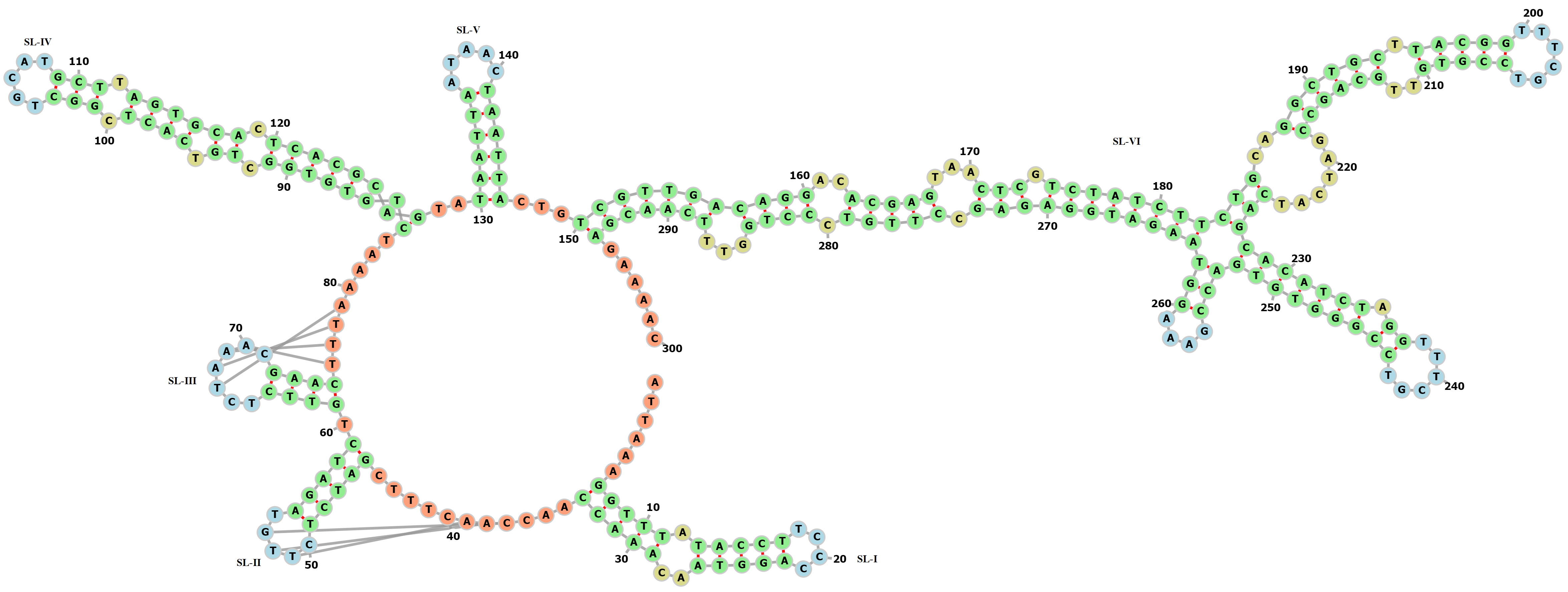

Supplement: Supplemental Information 4 — >5′-UTR (location:1..300) ATTAAAGGTTTATACCTTCCCAGGTAACAAACCAACCAACTTTCGATCTCTTGTAGATCTGTTCTCTAAACGAACTTTAAAATCTGTGTGGCTGTCACTCGGCTGCATGCTTAGTGCACTCACGCAGTATAATTAATAACTAATTACTGTCGTTGACAGGACACGAGTAACTCGTCTATCTTCTGCAGGCTGCTTACGGTTTCGTCCGTGTTGCAGCCGATCATCAGCACATCTAGGTTTCGTCCGGGTGTGACCGAAAGGTAAGATGGAGAGCCTTGTCCCTGGTTTCAACGAGAAAAC (((((.(((((....)))))..)))))...[[[.....(((((.]]].))))).((((..[[[[.))))]]]]....((((((((.((.((((.(((.....))).)))))).))))))))..((((((.....))))))...(((((((((((..(((((...(((.(((((((((((..((((((.(((((......)))))..))))))......)))(((((((.((......)))))))))(((....)))))))))))))).))))).))))...)))))))...... [file peerj-12-16962-s004.png]

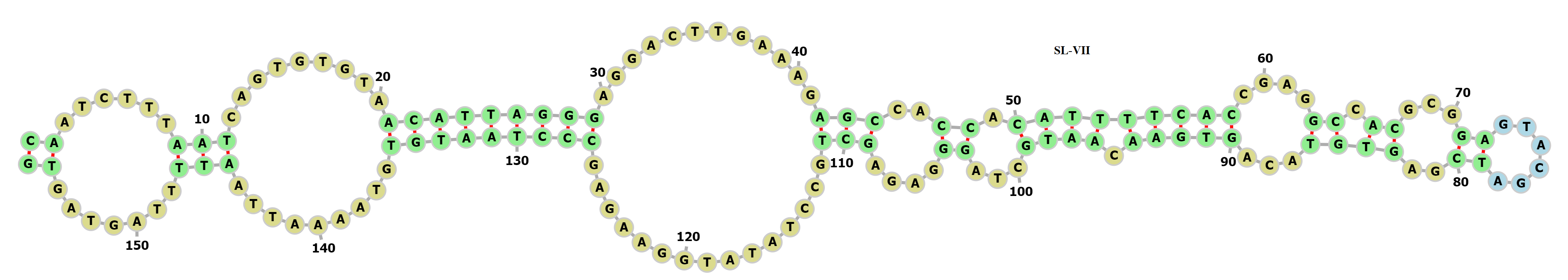

Supplement: Supplemental Information 5 — >3′-UTR (location: 29,675..29830) CAATCTTTAATCAGTGTGTAACATTAGGGAGGACTTGAAAGAGCCACCACATTTTCACCGAGGCCACGCGGAGTACGATCGAGTGTACAGTGAACAATGCTAGGGAGAGCTGCCTATATGGAAGAGCCCTAATGTGTAAAATTAATTTTAGTAGTG (((((((((((((((((..((.(((((((((....((.((...((......))..))))...))))).))))...))....)))...............))))))))).........))).......)) [file peerj-12-16962-s005.png]

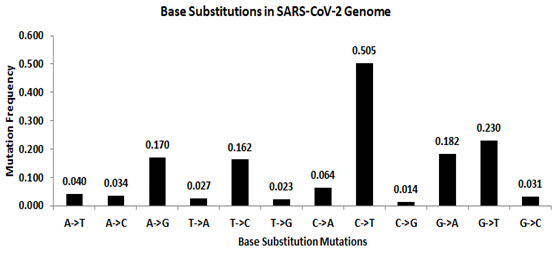

Supplement: Supplemental Information 6 — The twelve possible base substitutions along the complete SARS-CoV-2 genome. The height of the vertical bars in the Y-axis represents twelve directional mutation frequency values. The X-axis represents twelve mutations. [file peerj-12-16962-s006.png]

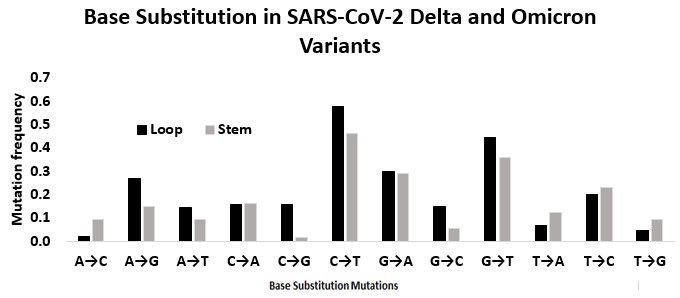

Supplement: Supplemental Information 7 — The height of the vertical bars in the Y-axis represents twelve directional mutation frequency values in the stem and loop regions. The X-axis represents twelve mutations. [file peerj-12-16962-s007.png]
